# Multi-omics integration reveals oral microbiota-metabolite-immune interactions in recurrent aphthous ulceration

**DOI:** 10.3389/fmicb.2026.1889297

**Published:** 2026-10-26

**Authors:** Cancan Shao, Haojie Wang, Jigao Li, Hongtao Guo, Xiaohong Gong, Ruilin Liu, Huifang He, Mingyi Shao

**Affiliations:** 1 Department of Rheumatology, The First Affiliated Hospital of Henan University of Chinese Medicine, Zhengzhou, Henan, China; 2 Department of Tuberculosis of Henan Provincial Chest Hospital, Zhengzhou, Henan, China; 3 Department of First Clinical School of Medicine of Henan University of Chinese Medicine, Zhengzhou, Henan, China; 4 Department of Gastroenterology, The First Affiliated Hospital of Henan University of Chinese Medicine, Zhengzhou, Henan, China

**Keywords:** immune dysregulation, metabolomics, oral microbiota, oxidative stress, recurrent aphthous ulceration

## Abstract

Recurrent aphthous ulceration (RAU) is a chronic inflammatory disease of the oral mucosa, but its underlying mechanisms remain unclear. In this study, integrated oral microbiome, serum metabolomics, and immune analyses were performed in 16 RAU patients and 16 healthy controls. 16S rRNA sequencing revealed suggestive compositional shifts in the oral microbiota of RAU patients, with nominal trends toward enrichment of *Fusobacterium*, *Moraxella*, and *Granulicatella* and depletion of *Treponema* and *Lancefieldella*. PICRUSt2 analysis indicated functional shifts associated with amino acid metabolism and inflammatory signaling. Untargeted metabolomics identified extensive metabolic remodeling, including altered gamma-glutamylcysteine, eicosapentaenoic acid (EPA), crocetin, pristanic acid, and cysteinyl-containing dipeptides, suggesting disturbances in oxidative stress regulation and inflammatory lipid metabolism. KEGG analysis further demonstrated enrichment of pathways related to cysteine and methionine metabolism, purine metabolism, taurine and hypotaurine metabolism, and primary bile acid biosynthesis. Immune profiling showed significant alterations in CD4^+^ T lymphocyte subsets, accompanied by elevated IL-1, IL-6, and TNF-α levels. Integrated correlation analysis revealed strong associations among differential microbial taxa, metabolites, and inflammatory cytokines. Collectively, these findings suggest that coordinated alterations in the oral microbiota, serum metabolites, and immune responses are associated with RAU, providing new insights into the biological features of RAU and potential therapeutic targets for further investigation.

## 1 Introduction

Recurrent aphthous ulceration (RAU), also referred to as recurrent aphthous stomatitis, is among the most prevalent inflammatory disorders of the oral mucosa and is clinically characterized by recurrent painful ulcerations surrounded by erythematous margins (Akintoye and Greenberg, 2014; Rivera, 2019). Although RAU is generally considered a self-limiting condition, its recurrent nature can substantially impair eating, speaking, and overall quality of life. Despite extensive investigation, the underlying pathogenesis of RAU has not yet been fully clarified. Current evidence suggests that RAU is a multifactorial disease involving genetic susceptibility, immune abnormalities, microbial dysbiosis, nutritional status, and environmental influences (Chiang et al., 2019; Edgar et al., 2017).

Over the past decade, the oral microbiota has emerged as an important factor in oral mucosal health and disease (Wade, 2013). The oral cavity contains one of the most complex microbial ecosystems in the human body, consisting of highly dynamic bacterial communities that interact closely with host epithelial and immune systems (Kilian et al., 2016; Lamont et al., 2018). Disturbance of this ecological balance, commonly referred to as dysbiosis, has been implicated in several inflammatory oral disorders. With the development of high-throughput 16S rRNA sequencing technologies, increasing studies have demonstrated that RAU is associated with alterations in oral microbial composition and community structure (Bankvall et al., 2014; Hijazi et al., 2015; Xiao et al., 2023). However, most previous studies have mainly focused on taxonomic changes, while the functional consequences of microbial dysbiosis in RAU remain insufficiently understood.

Metabolomics provides a powerful approach for characterizing downstream functional changes associated with both host physiology and microbial activity. Small-molecule metabolites represent the end products of cellular and microbial metabolic processes and can therefore reflect real-time biological status (Nicholson et al., 2012). In recent years, untargeted metabolomics has been increasingly applied to investigate inflammatory and immune-related diseases because it enables systematic identification of metabolic alterations and disturbed biochemical pathways (Wishart, 2019). Emerging evidence indicates that oxidative stress, amino acid metabolism, and lipid metabolic reshaping are closely associated with mucosal inflammation and epithelial barrier dysfunction (Estornut et al., 2024; Ghasemi et al., 2023; Zheng et al., 2023). Nevertheless, metabolomic features associated with RAU, particularly those potentially linked to oral microbial dysbiosis, remain poorly characterized.

Immune dysregulation is also considered a critical component in the development of RAU. Previous studies have reported abnormalities in both innate and adaptive immune responses, including altered T-cell subsets and increased production of pro-inflammatory cytokines (Bazrafshani et al., 2002; Lewkowicz et al., 2011; Slebioda et al., 2014). Excessive inflammatory activation may contribute to mucosal tissue injury and delayed ulcer healing. In this context, assessment of immune cell populations and inflammatory mediators may help to better understand the immunopathological mechanisms underlying RAU.

Importantly, growing evidence suggests that host immunity, microbial communities, and metabolic activities are not independent processes but are closely interconnected (Fan et al., 2018; Integrative HMP (iHMP) Research Network Consortium, 2019). Microbial dysbiosis may influence host immune responses through metabolite production, while inflammatory conditions can in turn reshape microbial composition and metabolic pathways (Zhao et al., 2025). However, studies integrating oral microbiome, metabolomics, and immune profiling in RAU are still very limited.

Therefore, in the present study, we performed a comprehensive multi-omics analysis integrating oral microbiota, serum metabolomics, and immune-related indicators in patients with RAU and healthy controls. Oral microbial composition was characterized using 16S rRNA sequencing, metabolic alterations were investigated through untargeted metabolomics, and immune status was evaluated using flow cytometry and ELISA. By integrating these datasets, we aimed to identify disease-associated microbial and metabolic signatures and to further explore the potential interactions among microbial dysbiosis, metabolic reprogramming, and immune imbalance in RAU. Our findings may provide new insights into the pathogenesis of RAU and contribute to the identification of potential biomarkers and therapeutic targets.

## 2 Materials and methods

### 2.1 Study design and participants

This prospective study consecutively enrolled patients with RAU from the Departments of Spleen-Stomach-Liver Diseases and Stomatology at The First Affiliated Hospital of Henan University of Chinese Medicine between October 1, 2025 and February 28, 2026. Age- and sex-matched healthy controls (HC) undergoing routine physical examination during the same period were recruited as the control group at a 1:1 ratio.

### 2.2 Inclusion and exclusion criteria

#### 2.2.1 RAU group

Inclusion criteria: (1) Age ≥ 18 years; (2) Diagnosis of RAU according to the Clinical Guidelines for Recurrent Aphthous Ulcers (2011) and Oral Mucosal Diseases (4th Edition); (3) Disease onset within 3 days prior to enrollment; (4) Ability to comply with study procedures.

Exclusion criteria: (1) Cognitive impairment, psychiatric disorders, or inability to complete study procedures; (2) Severe systemic diseases, including cardiovascular, hepatic, renal, hematological, or pulmonary disorders; (3) Pregnancy or lactation; (4) Use of antibiotics, probiotics, immunomodulators, or anti-inflammatory drugs within 4 weeks prior to enrollment.

#### 2.2.2 Control group

Inclusion criteria: (1) No history of RAU or other systemic diseases (e.g., autoimmune diseases, cardiovascular disease, hypertension, diabetes); (2) No long-term use of anti-inflammatory drugs, immunomodulators, probiotics, or probiotic-containing foods prior to enrollment; (3) Ability to comply with study procedures.

Exclusion criteria: (1) Presence of chronic diseases such as diabetes, coronary artery disease, atrial fibrillation, heart failure, chronic kidney disease, or malignancy; (2) Pregnancy or lactation.

### 2.3 Baseline data collection

Baseline demographic and clinical characteristics, including age, sex and body mass index (BMI) were collected at enrollment.

### 2.4 Sample collection and processing

Fasting peripheral blood and unstimulated saliva samples were collected from all participants in the early morning after at least 8 h of fasting. Blood samples were drawn into serum separation tubes, allowed to clot at room temperature for 60 min, and centrifuged at 1, 600 × *g* for 10 min at 4 °C within 30 min of collection to obtain serum, which was aliquoted and stored at −80 °C. Saliva samples (1–2 mL) were collected into sterile tubes and immediately stored at −80 °C. All samples were processed under standardized conditions and used for subsequent analyses, including immune profiling, cytokine quantification, metabolomics, and oral microbiota sequencing.

### 2.5 Flow cytometry analysis of T cell subsets

Peripheral blood mononuclear cells (PBMCs) were isolated by density gradient centrifugation and stained with fluorochrome-conjugated monoclonal antibodies targeting surface markers of CD3^+^, CD4^+^, and CD8^+^ T-lymphocyte subsets according to the manufacturer’s instructions. Flow cytometric analysis was performed using a Beckman Coulter flow cytometer (Shanghai, China) to quantify T lymphocyte subsets, including CD3^+^, CD4^+^, and CD8^+^ T lymphocyte subsets. The CD4^+^/CD8^+^ ratio was calculated, and data were analyzed using FlowJo software to assess immune cell distribution.

### 2.6 Measurement of inflammatory cytokines

Serum levels of inflammatory cytokines, including interleukin-1 (IL-1), interleukin-6 (IL-6), and tumor necrosis factor-α (TNF-α), were measured using enzyme-linked immunosorbent assay kits according to the manufacturers’ protocols. Absorbance was read using a microplate reader (Multiskan FC, Thermo Fisher Scientific, China), and cytokine concentrations were calculated based on standard curves generated for each analyte.

### 2.7 Untargeted metabolomics analysis

Serum samples were thawed on ice and metabolites were extracted by adding 120 μL of pre-chilled 50% methanol to 20 μL of serum, followed by vortexing for 1 min, incubation at room temperature for 10 min, and centrifugation at 4,000 × g for 20 min. The supernatant was collected for analysis. Quality control (QC) samples were prepared by pooling equal volumes from all samples. Metabolomic profiling was performed using a Vanquish Flex ultra-high-performance liquid chromatography system (Thermo Fisher Scientific, United States) coupled with a Q Exactive high-resolution mass spectrometer (Thermo Fisher Scientific, United States). Chromatographic separation was achieved using an ACQUITY UPLC T3 column (100 mm × 2.1 mm, 1.8 μm; Waters, United States) at 35 °C with a flow rate of 0.4 mL/min, using a mobile phase consisting of water with 0.1% formic acid (solvent A) and acetonitrile with 0.1% formic acid (solvent B). The gradient elution program was set as follows: 0–0.5 min, 5% B; 0.6–7.0 min, 5–100% B; 7.1–7.9 min, 100% B; 8.0–8.1 min, 100–5% B; and 8.2–10.0 min, 5% B. Mass spectrometry data were acquired in both positive and negative ion modes.

Raw metabolomics data were processed using XCMS software for peak detection, alignment, and normalization. Multivariate statistical analyses, including principal component analysis (PCA) and partial least squares discriminant analysis (PLS-DA), were performed to evaluate data quality and group separation. Differential metabolites were identified based on variable importance in projection (Variable Importance in Projection (VIP) > 1.0), fold change (Fold Change (FC) > 1.5 or FC < 0.667), and *p*-value < 0.05 (Student’s *t*-test). Metabolite identification was conducted by comparison with the Human Metabolome Database and PubChem, and exogenous metabolites were excluded. Pathway enrichment analysis was performed using KEGG via MetaboAnalyst (version 6.0). Visualization, including volcano plots and heatmaps, as well as receiver operating characteristic (ROC) curve analysis for biomarker evaluation, was conducted using R software (version 4.1.3) (Note: Metabolites detected by untargeted LC–MS/MS were putatively annotated based on accurate precursor mass, available MS/MS fragmentation information, and database matching against HMDB and PubChem. Because authentic standards were not systematically used for metabolite confirmation, the reported metabolite identities should be interpreted as putative annotations rather than definitive compound identifications).

### 2.8 16S rRNA sequencing and microbiota analysis

Microbial genomic DNA was extracted from saliva samples, and the V3–V4 regions of the bacterial 16S rRNA gene were amplified and sequenced using the Illumina MiSeq platform. Paired-end reads were merged based on overlap and subjected to quality filtering to remove low-quality reads, chimeras, and adapter contamination. Amplicon sequence variants (ASVs) were generated using the UNOISE3 algorithm implemented in USEARCH (v10.0), and downstream bioinformatic analyses were performed with QIIME2. Alpha diversity indices, including ACE, Chao1, Shannon, and Simpson indices, were calculated to assess microbial richness and diversity. Beta diversity was evaluated using unweighted and weighted UniFrac distances, followed by multivariate analyses such as PCA, principal coordinates analysis (PCoA), and non-metric multidimensional scaling (NMDS) to explore differences in microbial community structure between groups. Group differences were statistically assessed using Adonis (PERMANOVA) and ANOSIM, and differential taxa were identified based on relative abundance comparisons.

To infer the functional potential of the microbial communities based on 16S rRNA gene sequencing data, Phylogenetic Investigation of Communities by Reconstruction of Unobserved States 2 (PICRUSt2) was employed following the framework described in Douglas et al. (2020). Briefly, ASVs obtained from 16S rRNA sequencing were used as input for downstream functional prediction. First, ASV sequences were aligned and placed into a reference phylogenetic tree containing genomes with known functional annotations using a phylogenetic placement algorithm. Subsequently, gene family copy numbers for each ASV were predicted using a hidden-state prediction (HSP) approach, which infers the genomic content of unobserved organisms based on their phylogenetic relationships with reference taxa. The predicted gene family abundances were then normalized by 16S rRNA gene copy number and combined with ASV abundance data across samples to generate sample-wise metagenome predictions. Finally, functional pathway abundances were inferred from gene family profiles using pathway reconstruction algorithms (e.g., MinPath), enabling the prediction of Kyoto Encyclopedia of Genes and Genomes (KEGG) orthologs and MetaCyc metabolic pathways (Note: PICRUSt2 was applied to predict the functional potential of oral microbial communities based on 16S rRNA sequencing data. Predicted KEGG orthologs and MetaCyc pathways were interpreted as potential microbial functional characteristics rather than experimentally validated functions).

### 2.9 Integrated multi-omics analysis

To explore the interactions among microbiota, metabolites, and immune parameters, correlation analyses were performed using Spearman’s rank correlation. Significant associations were visualized using correlation heatmaps and network analysis. Key microbial taxa and metabolites were further linked to immune indicators to construct a microbiota-metabolite-immune interaction network. Correlation analysis was performed using the OmicStudio tools at https://www.omicstudio.cn/tool.

### 2.10 Statistical analysis

All statistical analyses were performed using IBM SPSS software (version 24.0). Data normality was assessed using the Kolmogorov–Smirnov test. Normally distributed data were expressed as mean ± standard deviation (SD) and compared using Student’s *t*-test, whereas non-normally distributed data were presented as median (interquartile range). The Wilcoxon rank-sum (Mann–Whitney *U*) test was used for two-group comparisons of non-normally distributed variables. For low-dimensional clinical and immune parameters, a raw *p*-value < 0.05 was considered statistically significant. For high-dimensional genus- and phylum-level microbiota differential abundance analyses, the Benjamini–Hochberg false-discovery-rate (FDR) procedure was applied to correct for multiple testing. Nominal raw *p*-values were reported alongside Benjamini–Hochberg adjusted *p*-values (FDR); taxa with FDR < 0.05 were defined as statistically significant after multiple-test correction.

## 3 Results

### 3.1 Baseline characteristics of study participants

A total of 32 participants were included in this study, comprising 16 patients with recurrent aphthous ulceration (RAU) and 16 age- and sex-matched healthy controls. As shown in Table 1, there were no significant differences between the two groups in terms of age, sex distribution, body mass index (BMI) (all *p*-value > 0.05), indicating good comparability between groups and minimizing potential confounding effects (Table 1).

**Table 1:** General information.

Group	Number of cases	Gender		Age (years)	Body mass index (kg/m^2^)
		Male	Female		
HC group	16	7	9	40.75 ± 3.74	24.3 ± 3.1
RAU group	16	9	7	35.44 ± 1.47	22.8 ± 2.7
*p*-value	–	0.481		0.204	0.152

### 3.2 Alterations in oral microbiota diversity and community structure in RAU

To evaluate changes in oral microbial diversity, alpha and beta diversity analyses were performed. Alpha diversity indices, including Shannon and Simpson, showed a decreasing trend in the RAU group compared with controls, although the differences did not reach statistical significance, suggesting a modest reduction in microbial richness and diversity (Figures 1A,B). The RAU group uniquely had 1,247 ASVs, the HC group uniquely had 1,117 ASVs, and the two groups shared 898 ASVs (Figure 1C). Beta diversity analysis based on unweighted UniFrac distances demonstrated a clear separation between RAU patients and healthy controls, as visualized by principal coordinate analysis (PCoA) and non-metric multidimensional scaling (NMDS) (Figures 1D,E). The species accumulation curve showed that when the sample size reached 36, the curve tended to flatten, and the number of newly detected species did not exhibit significant fluctuations, indicating that a sample size of 36 was appropriate (Figure 1F).

**Figure 1:**
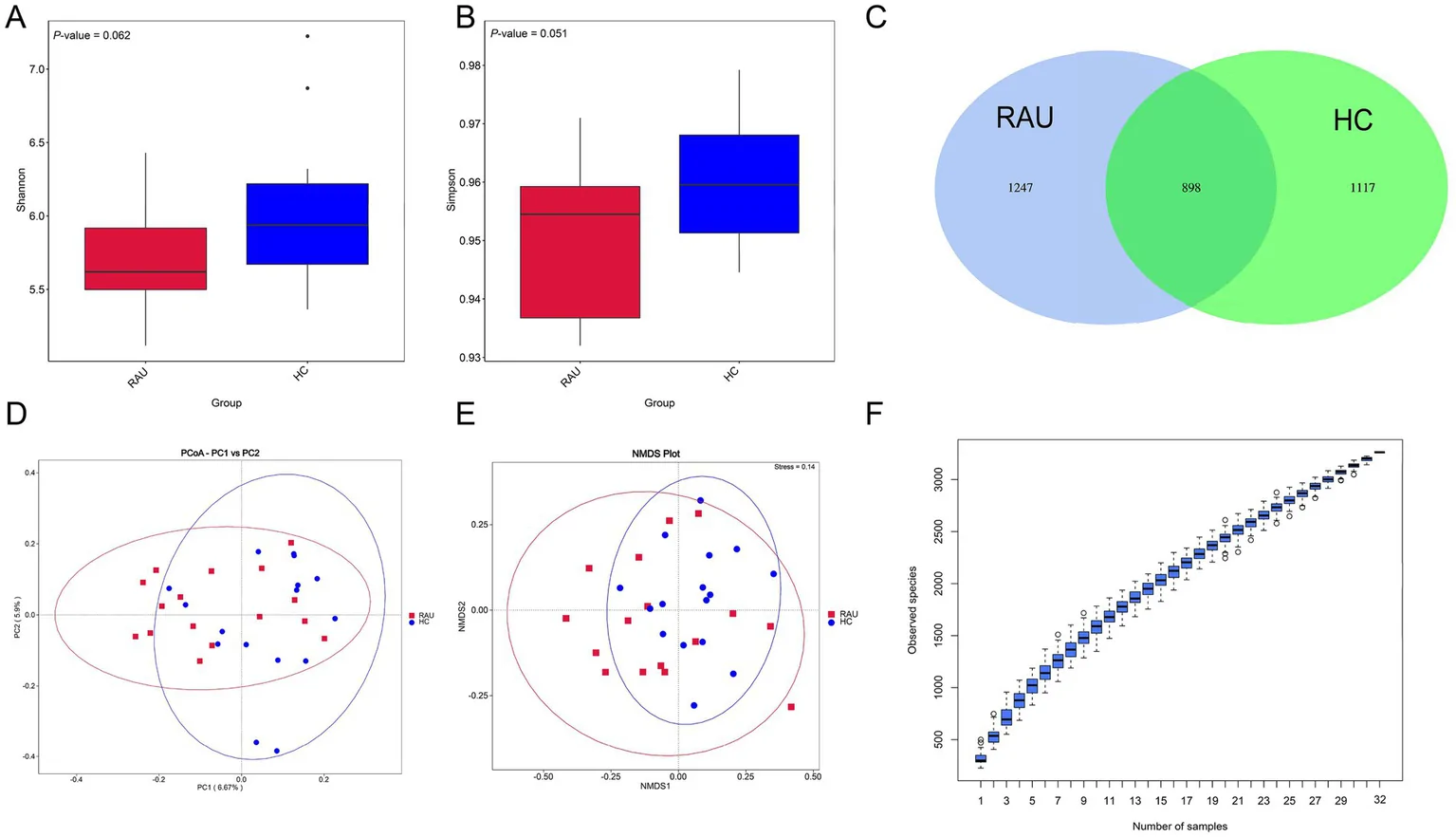
Alterations in oral microbial diversity and community structure in RAU. **(A)** Shannon index comparing oral microbial alpha diversity between healthy controls (HC) and RAU patients. **(B)** Simpson index comparing oral microbial alpha diversity between groups. **(C)** Venn diagram showing shared and unique ASVs between HC and RAU groups. **(D)** Principal coordinate analysis (PCoA) based on unweighted UniFrac distances demonstrating beta diversity differences between groups. **(E)** Non-metric multidimensional scaling (NMDS) analysis of oral microbial community structure between HC and RAU samples. **(F)** Species accumulation curve indicating sequencing depth and adequacy of sample size.

### 3.3 Differential microbial taxa associated with RAU

The composition and structure of the oral microbiota were compared between the HC group and RAU patients to identify disease-associated microbial signatures. At the phylum level, the oral microbiota of both groups was dominated by Bacteroidota, Pseudomonadota and Bacillota (Figure 2A). No taxa reached statistical significance after Benjamini-Hochberg multiple-testing correction at this taxonomic level, although suggestive abundance trends were observed: Fusobacteriota and Patescibacteria tended to be increased, whereas Spirochaetota tended to be decreased in the RAU group, implying a potential global shift in microbial community structure under disease conditions (Figure 2A). Among the detected taxa, *Fusobacterium* and *Moraxella* showed suggestive trends of higher abundance in the RAU group, while *Treponema* displayed a suggestive trend of lower abundance relative to healthy controls (Figure 2B). These alterations hint at a possible transition toward a dysbiotic microbial state characterized by the expansion of potentially pro-inflammatory taxa and the depletion of putative commensal bacteria. To further evaluate the contribution of individual taxa to the overall microbial dissimilarity between groups, Similarity Percentage (SIMPER) analysis was performed. The results demonstrated that a limited number of genera accounted for the majority of inter-group variation. Notably, *Neisseria*, *Prevotella* and *Haemophilus* were identified as major contributors to the observed dissimilarity driven by their altered abundance in RAU samples (Figure 2C). Differential abundance analysis visualized via a volcano plot further illustrated these exploratory findings. Specifically, *Granulicatella* showed a nominal trend of higher abundance in the RAU group, while *Lancefieldella* showed a nominal trend of lower abundance (Figure 2D). These taxa represent potential microbial signatures associated with disease status. The consistency of these abundance patterns was further supported by heatmap-based hierarchical clustering, which largely separated HC and RAU samples into distinct clusters based on genus-level abundance profiles. In this analysis, *Granulicatella* tended to exhibit higher abundance across RAU samples (Figure 2E). To quantitatively visualize these nominal differences, bar-plot analysis was performed for representative taxa highlighted in the volcano plot. Nominal raw *p*-values < 0.05 for *Granulicatella* and *Lancefieldella*; however, neither taxon remained significant following Benjamini-Hochberg FDR multiple-testing correction (Figure 2F and Supplementary Table S1).

**Figure 2:**
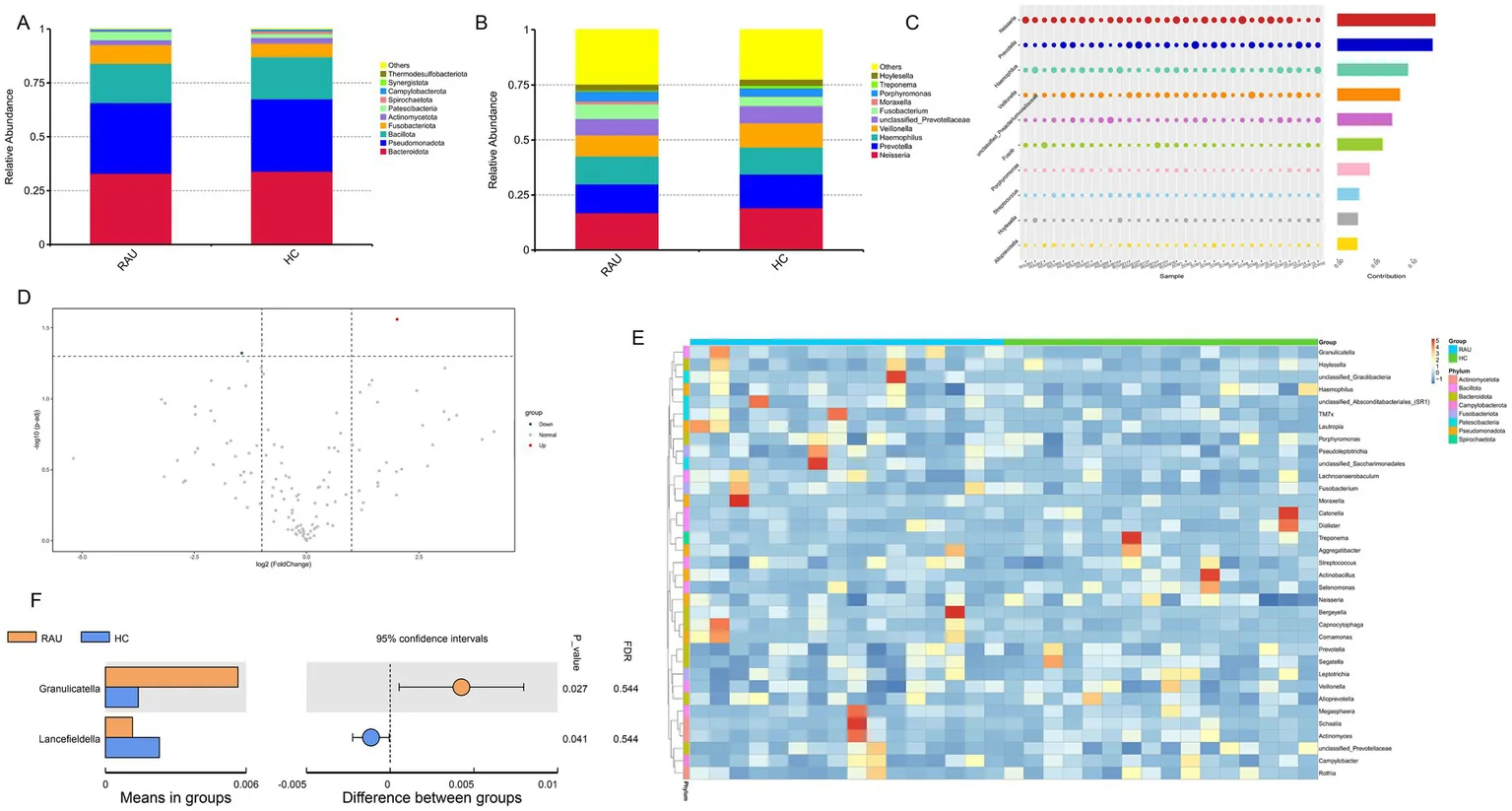
Differential oral microbial taxa associated with RAU. **(A)** Relative-abundance profiles of the top 10 most abundant phyla across groups. **(B)** Relative-abundance profiles of the top 10 most abundant genera across groups. **(C)** Similarity percentage (Simper) analysis showing major bacterial genera contributing to inter-group microbial dissimilarity. **(D)** Volcano plot showing significantly altered microbial taxa between HC and RAU groups. Red dots indicate significantly enriched taxa, whereas blue dots indicate significantly decreased taxa in RAU samples. **(E)** Heatmap-based hierarchical clustering analysis of differential genus-level microbial profiles between groups. **(F)** Bar plot analysis of representative differential taxa identified from volcano plot analysis, including *Granulicatella* and *Lancefieldella*.

### 3.4 Functional prediction of oral microbiota using PICRUSt2

To explore the functional implications of microbial alterations, PICRUSt2 was applied to predict metagenomic functions based on 16S rRNA gene data. Functional profiling revealed differences in predicted gene families and metabolic pathways between RAU patients and controls. PCA of the predicted functional abundance profiles revealed a clear separation between the RAU and healthy control groups, indicating substantial differences in the functional potential of the oral microbial communities between the two conditions (Figure 3A). To further characterize the distribution of predicted functional genes among groups, Venn diagram analysis was performed to evaluate shared and unique functional features. The results demonstrated that the RAU group possessed 137 unique predicted functions, whereas the healthy control group (HC) contained 11 unique functions. In addition, a total of 6,118 predicted functions were shared between the two groups (Figure 3B). At the pathway level, Kyoto Encyclopedia of Genes and Genomes (KEGG) analysis demonstrated that pathways related to amino acid metabolism, carbohydrate metabolism, and inflammatory signaling were differentially enriched. Notably, several pathways involved in signaling molecules and interaction infectious showed altered abundance in the RAU group, indicating that microbial dysbiosis may contribute to disease development through functional reprogramming rather than solely compositional changes (Figure 3C).

**Figure 3:**
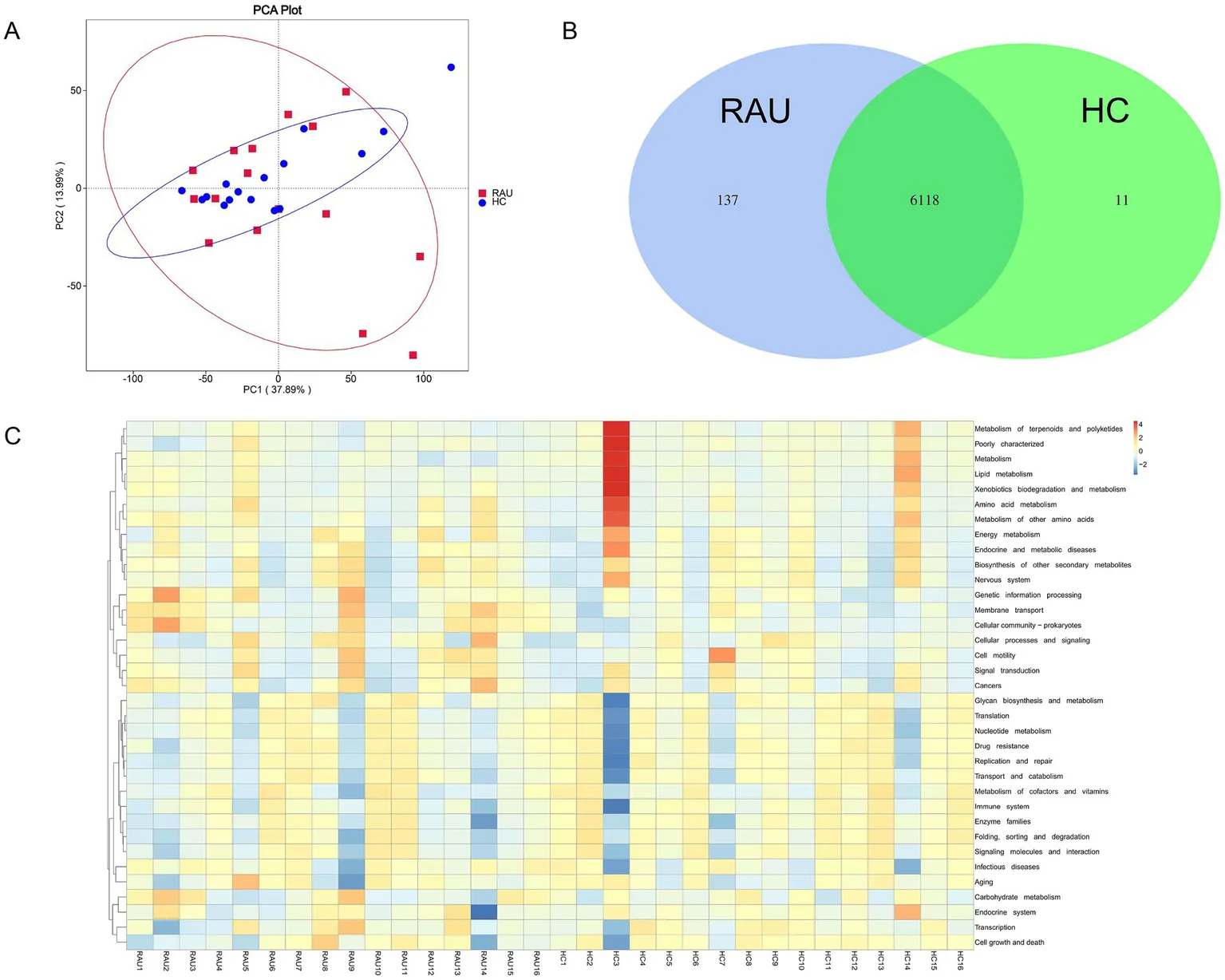
Functional prediction of oral microbiota based on PICRUSt2 analysis. **(A)** Principal component analysis (PCA) of predicted functional abundance profiles between HC and RAU groups. **(B)** Venn diagram showing shared and unique predicted microbial functions between groups. **(C)** KEGG functional pathway enrichment analysis of predicted microbial metabolic pathways.

### 3.5 Global metabolomic profiling of serum samples

Untargeted serum metabolomics analysis was performed in both negative and positive ion modes to comprehensively characterize metabolic alterations associated with RAU. In the negative ion mode, metabolite annotation revealed that the detected compounds were mainly classified into Lipids and lipid-like molecules (44.30%), Organoheterocyclic compounds (14.44%) and Organic acids and derivatives (12.18%), indicating substantial coverage of fatty acid- and redox-related metabolites (Figure 4A). Principal component analysis (PCA) demonstrated a discernible separation trend between the RAU and healthy control groups, suggesting disease-associated metabolic alterations despite partial overlap between samples (Figure 4B). Quality control (QC) samples clustered tightly in the PCA score plot, indicating excellent analytical reproducibility and instrument stability. Consistently, correlation analysis among QC samples showed high Pearson correlation coefficients, further confirming the reliability of metabolite detection in the negative ion mode (Figure 4C). In the positive ion mode, the identified metabolites were primarily enriched in Lipids and lipid-like molecules (37.54%), Organic acids and derivatives (18.53%) and Organoheterocyclic compounds (17.39%) (Figure 4D). Similar to the negative ion mode, PCA analysis revealed an overall separation tendency between RAU and control samples, indicating systematic metabolic differences associated with disease status (Figure 4E). Moreover, QC samples displayed tight clustering and strong inter-sample correlations, demonstrating robust platform stability and high-quality metabolomics data in the positive ion mode (Figure 4F).

**Figure 4:**
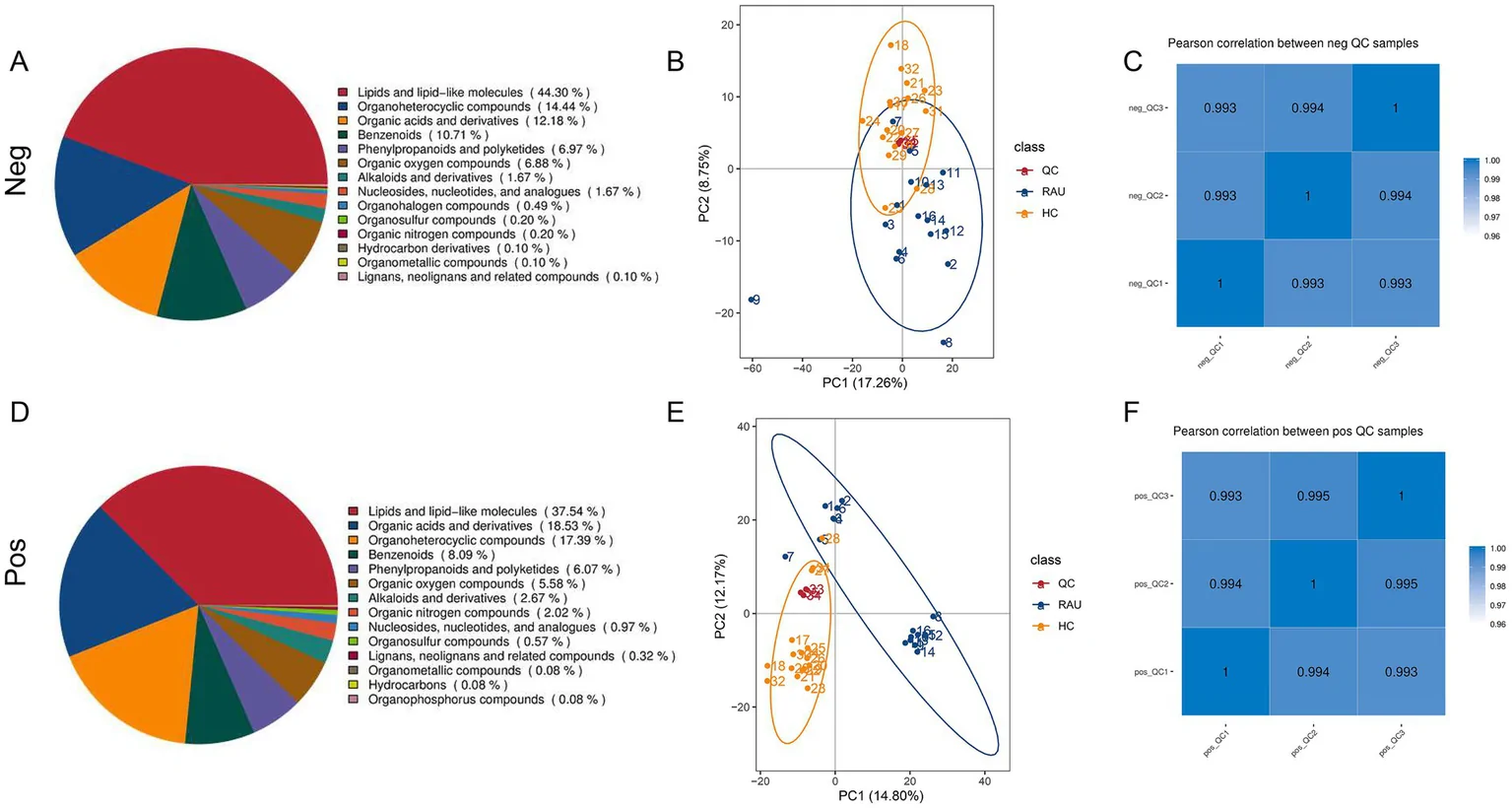
Global metabolomic profiling of serum samples in RAU. **(A)** Classification of identified metabolites in negative ion mode. **(B)** PCA score plot of serum metabolites detected in negative ion mode. **(C)** Pearson correlation heatmap of quality control (QC) samples in negative ion mode. **(D)** Classification of identified metabolites in positive ion mode. **(E)** PCA score plot of serum metabolites detected in positive ion mode. **(F)** Pearson correlation heatmap of QC samples in positive ion mode.

### 3.6 Identification and characterization of differential metabolites

To further identify disease-associated metabolites, differential metabolite analysis was conducted separately for the negative and positive ion mode datasets. In the negative ion mode, based on the predefined screening criteria (VIP > 1.0, FC > 1.5 or <0.667, and FDR < 0.05), 55 significantly altered metabolites were identified compared with the control group, among which 16 were significantly up-regulated and 39 significantly down-regulated (Figure 5A). Stick plot analysis further illustrated the overall abundance changes of these metabolites between groups (Figure 5B). In the positive ion mode, volcano plot analysis identified 204 significantly altered metabolites involved in inflammatory regulation, oxidative stress, and lipid metabolism (Figure 5C). Stick plot analysis demonstrated obvious abundance differences of these metabolites between groups (Figure 5D and Supplementary Table S2).

**Figure 5:**
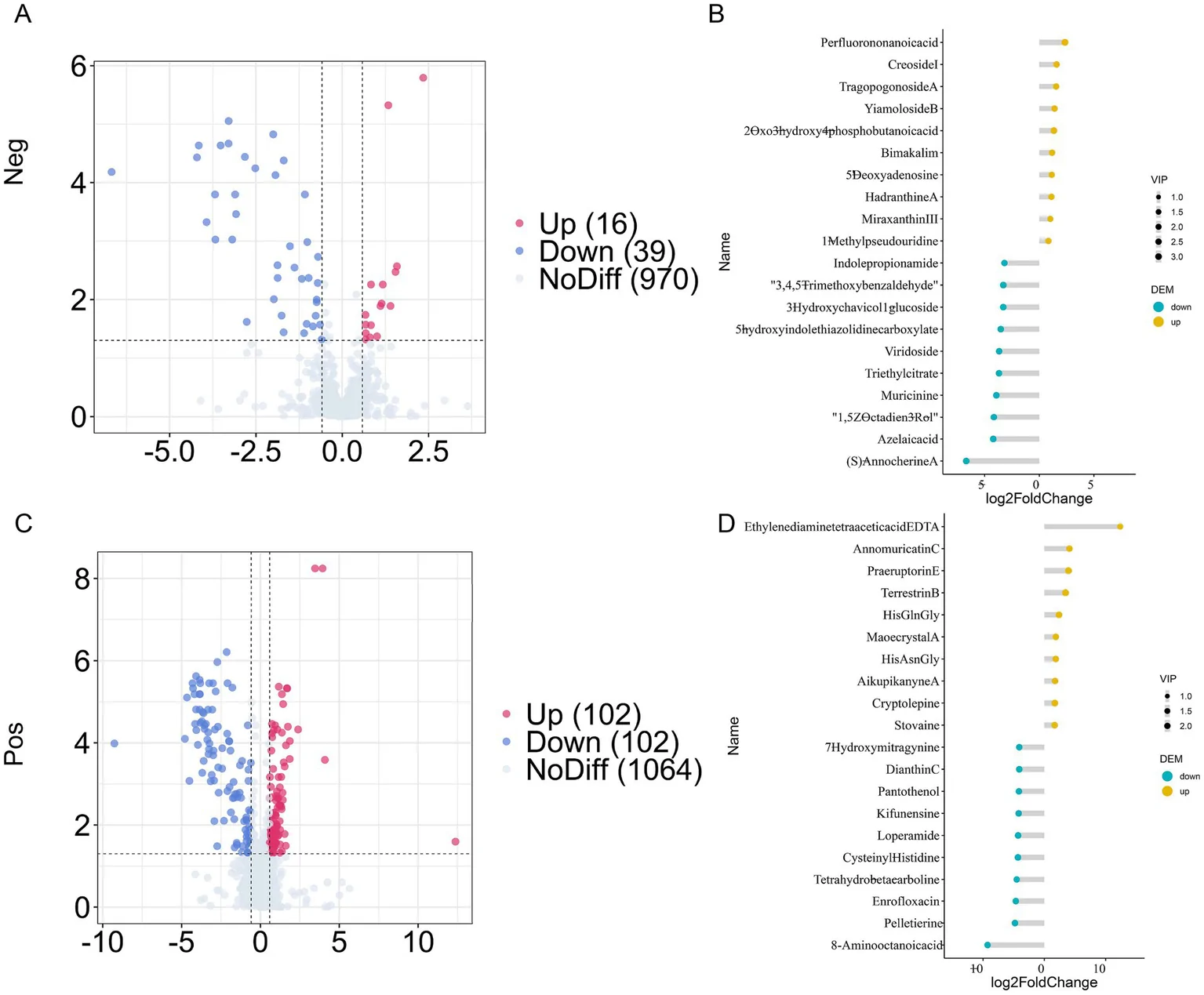
Identification of differential metabolites between RAU patients and healthy controls. **(A)** Volcano plot showing significantly altered metabolites in negative ion mode based on *VIP* > 1.0, *FC* > 1.5 or < 0.667, and *FDR* < 0.05. (Red dots represent upregulated metabolites, and blue dots represent downregulated metabolites in the RAU group). **(B)** Stick plot illustrating relative abundance changes of differential metabolites in negative ion mode. **(C)** Volcano plot showing significantly altered metabolites in positive ion mode. **(D)** Stick plot illustrating relative abundance changes of differential metabolites in positive ion mode.

Hierarchical clustering heatmap analysis clearly separated RAU samples from healthy controls, indicating consistent disease-associated metabolic signatures in the negative ion mode (Figure 6A). In addition, boxplot analysis of representative metabolites confirmed significant alterations in gamma-glutamylcysteine, pristanic acid, undecanedioic acid, and isethionic acid, which were mainly associated with glutathione metabolism, lipid oxidation, and microbial-associated metabolic processes (Figures 6B–E). Heatmap-based hierarchical clustering further confirmed distinct metabolic patterns between RAU and healthy controls in the positive ion mode (Figure 6F). Moreover, boxplot analysis showed that eicosapentaenoic acid (EPA), crocetin, cortisone acetate, and cysteinyl-histidine dipeptides metabolites exhibited significant changes in RAU patients, suggesting impaired inflammation resolution and disrupted redox homeostasis (Figures 6G–J).

**Figure 6:**
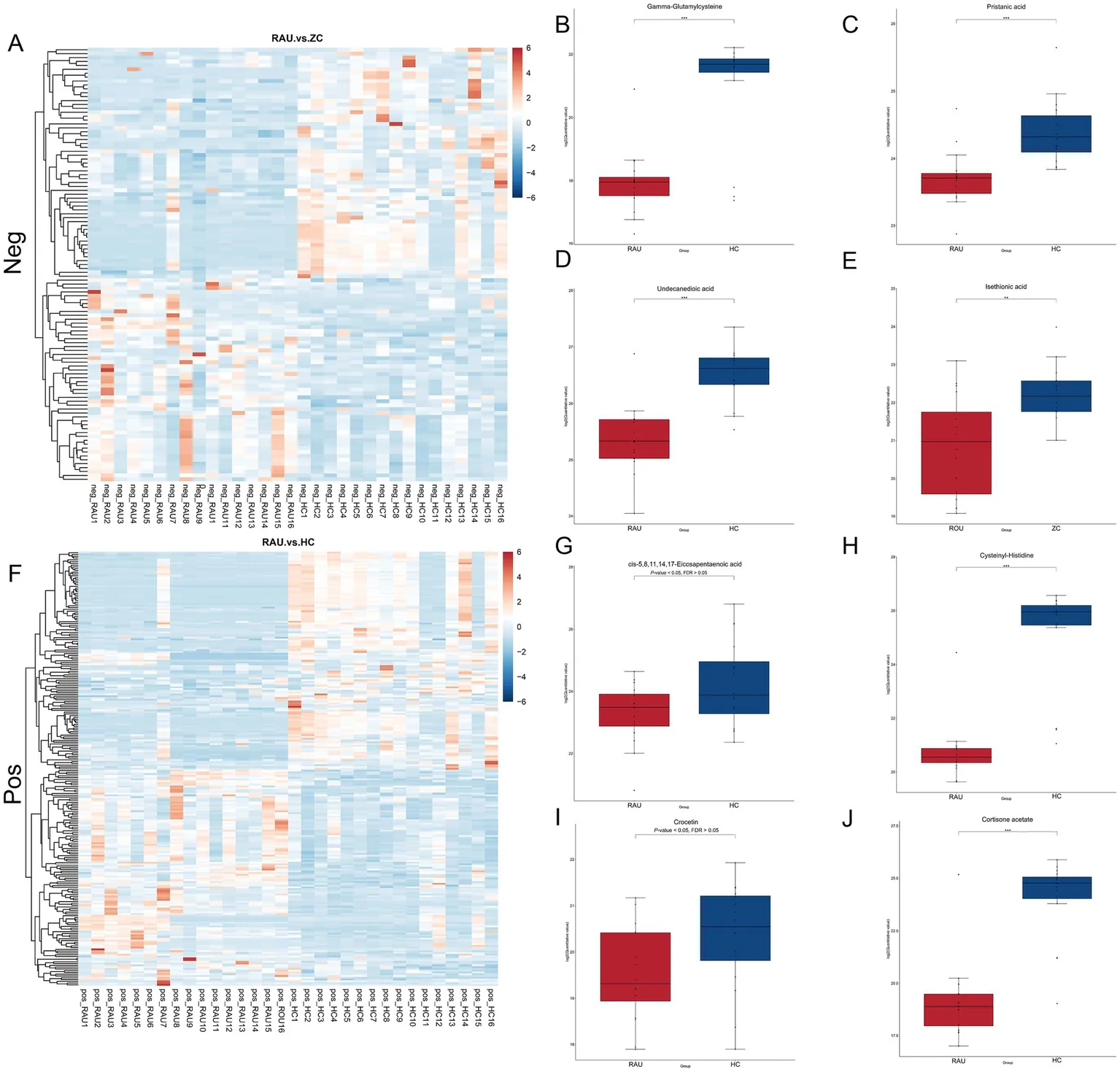
Characterization of representative differential metabolites in RAU. **(A)** Heatmap-based hierarchical clustering analysis of differential metabolites in negative ion mode. **(B–E)** Boxplot analysis of representative metabolites in negative ion mode, including gamma-glutamylcysteine, pristanic acid, undecanedioic acid, and isethionic acid. **(F)** Heatmap-based hierarchical clustering analysis of differential metabolites in positive ion mode. **(G–J)** Boxplot analysis of representative metabolites in positive ion mode, including EPA, crocetin, cortisone acetate, and cysteinyl-histidine dipeptides.

### 3.7 KEGG pathway enrichment and metabolic network analysis

To further investigate the biological significance of the identified differential metabolites, KEGG pathway enrichment analysis (Based on PICRUSt2 functional predictions) was performed separately for the negative and positive ion mode datasets. In the negative ion mode, KEGG enrichment bubble plot analysis revealed that several pathways were enriched in RAU patients, including caffeine metabolism, drug metabolism-other enzymes, cysteine and methionine metabolism, β-alanine metabolism, and nicotinate and nicotinamide metabolism. Among these, caffeine metabolism showed the strongest enrichment signal, involving metabolites such as 1,7-dimethyluric acid, theophylline, and 1-methylxanthine (Figure 7A). In addition, pathways associated with amino acid metabolism and redox regulation, particularly cysteine and methionine metabolism, were also enriched, suggesting disturbances in antioxidant defense and glutathione-related metabolic processes in RAU. KEGG regulatory network analysis in the negative ion mode further demonstrated extensive interactions among pathways related to nucleotide metabolism, alanine/aspartate/glutamate metabolism, pyrimidine metabolism, and drug metabolism pathways (Figure 7B). Notably, alanine, aspartate and glutamate metabolism occupied an important position within the regulatory network, supporting the presence of amino acid metabolic remodeling under inflammatory conditions.

**Figure 7:**
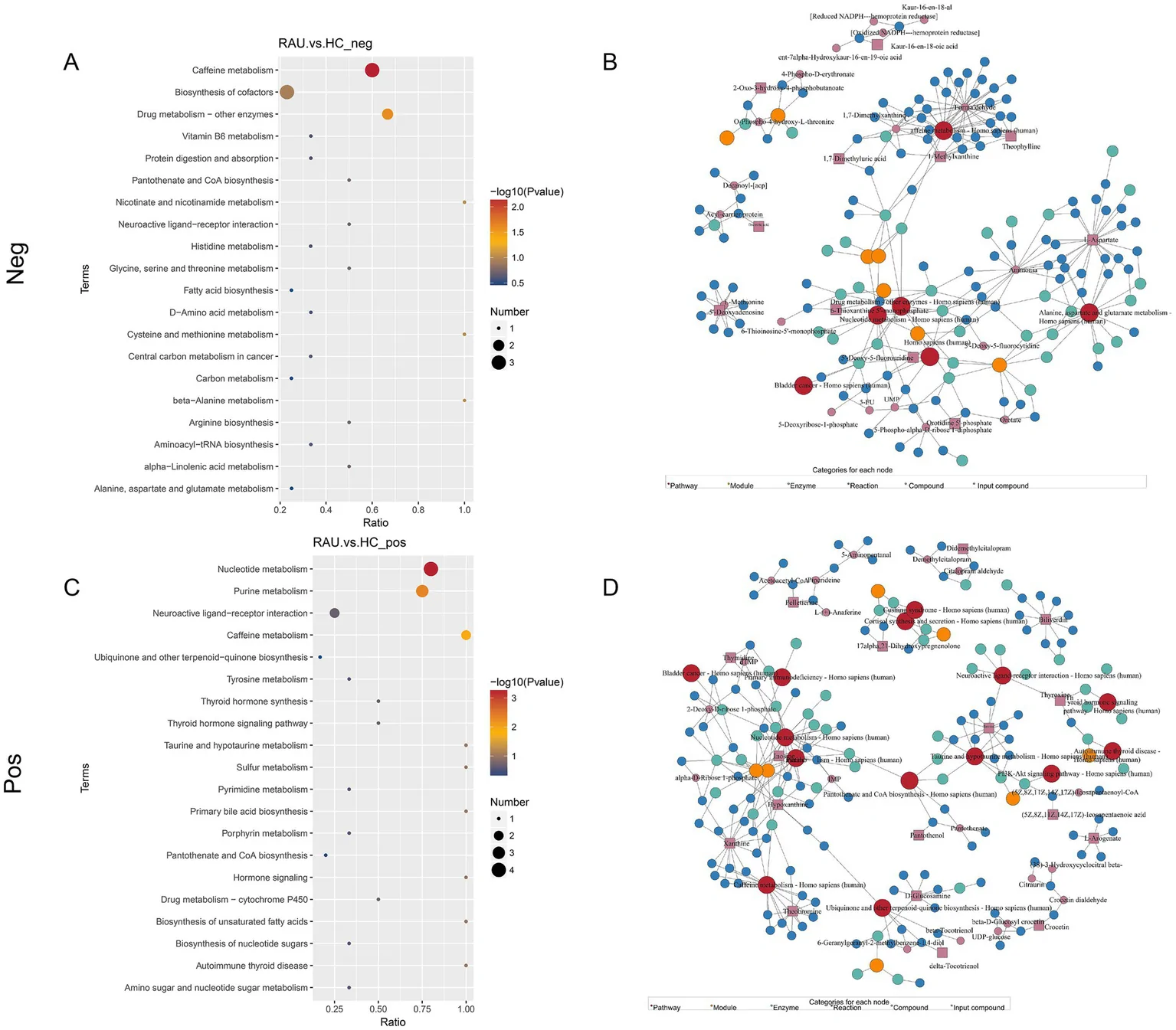
KEGG pathway enrichment and metabolic network analysis of differential metabolites. **(A)** KEGG enrichment bubble plot analysis of significantly altered metabolic pathways in negative ion mode. **(B)** KEGG regulatory network analysis of enriched metabolic pathways in negative ion mode. **(C)** KEGG enrichment bubble plot analysis of significantly altered metabolic pathways in positive ion mode. **(D)** KEGG regulatory network analysis of enriched metabolic pathways in positive ion mode. Bubble size represents metabolite number enriched in each pathway, and bubble color indicates enrichment significance.

In the positive ion mode, KEGG enrichment analysis identified several altered pathways, including nucleotide metabolism, purine metabolism, caffeine metabolism, primary bile acid biosynthesis, and taurine and hypotaurine metabolism. Among these pathways, nucleotide metabolism and purine metabolism exhibited the highest enrichment levels and involved metabolites such as hypoxanthine, xanthine, thymidine, and inosine, indicating enhanced nucleotide turnover and metabolic stress in RAU patients. In addition, enrichment of taurine and hypotaurine metabolism suggested alterations in antioxidant and inflammatory regulatory pathways, while the identification of primary bile acid biosynthesis implied potential interactions between systemic metabolism and inflammatory status (Figure 7C). Consistently, KEGG regulatory network analysis in the positive ion mode revealed close interactions among pathways involved in ubiquinone and other terpenoid-quinone biosynthesis, purine metabolism, taurine and hypotaurine metabolism, pantothenate and CoA biosynthesis, and caffeine metabolism. Several pathways associated with energy metabolism, oxidative stress regulation, and inflammatory lipid metabolism occupied central positions within the network, indicating coordinated metabolic reprogramming in RAU (Figure 7D).

### 3.8 Alterations in T cell subsets and inflammatory cytokines in RAU

Flow cytometry analysis revealed a significant reduction in CD4^+^ T-cell levels in RAU patients (*p*-value < 0.05), whereas CD8^+^ T-cell levels and the CD4^+^/CD8^+^ ratio did not differ significantly between groups (*p*-value > 0.05). These findings suggest altered T-cell characteristics in RAU rather than a generalized T-cell imbalance (Table 2). In addition, serum levels of pro-inflammatory cytokines, including IL-1, IL-6, and TNF-α, were significantly elevated in the RAU group (*p*-value < 0.05), reflecting a systemic inflammatory state. These results support the notion that immune dysregulation plays a central role in RAU pathogenesis (Table 3).

**Table 2:** Comparison of T lymphocyte subset indices between the two groups.

Group	Number of cases	CD3^+^ (%)	CD4^+^ (%)	CD8^+^ (%)	CD4^+^/CD8^+^
HC group	16	70.83 ± 7.28	37.89 ± 9.29	31.65 ± 5.35	1.25 ± 0.43
RAU group	16	69.70 ± 10.53	31.71 ± 5.47	34.71 ± 11.98	1.11 ± 0.70
*P*-value		0.725	0.030	0.360	0.495

**Table 3:** Comparison of inflammatory cytokine levels between the two groups.

Group	Number of cases	IL-1 (pg/ml)	IL-6 (pg/ml)	TNF-α (pg/ml)
HC group	16	0.95 ± 0.42	0.54 ± 0.20	1.28 ± 0.58
RAU group	16	1.56 ± 0.73	0.84 ± 0.40	2.35 ± 1.07
*P*-value		0.042	0.015	0.006

### 3.9 Integrated analysis reveals microbiota-metabolite-immune interactions

To investigate potential interactions between oral microbiota and metabolic alterations, Spearman correlation analysis was performed between differential microbial taxa and metabolites. Several bacterial genera showed significant correlations with disease-associated metabolites, suggesting potential links between microbial dysbiosis and metabolic disturbances in RAU. Correlation network analysis demonstrated extensive associations among oral microbial composition, serum metabolites, and inflammatory immune markers, indicating the presence of coordinated host–microbe interactions in RAU. Among the differential microbial taxa, *Granulicatella* exhibited significant positive correlations with pro-inflammatory cytokines, including IL-1, IL-6, and TNF-α. In contrast, *Lancefieldella* showed negative correlations with inflammatory cytokines and positive associations with antioxidant-related metabolites. Notably, several oxidative stress- and lipid metabolism-related metabolites displayed strong associations with both microbial taxa and immune parameters. Gamma-Glutamylcysteine and cysteinyl-containing dipeptides were negatively correlated with inflammatory-associated genera and pro-inflammatory cytokines, while showing positive correlations with CD4^+^ T-cell-associated immune balance indicators. Similarly, eicosapentaenoic acid (EPA) and crocetin exhibited negative correlations with inflammatory cytokines and positive associations with potentially beneficial microbial taxa, supporting their roles in inflammation resolution and redox regulation. In addition, metabolites involved in purine metabolism, taurine and hypotaurine metabolism, and cysteine and methionine metabolism were closely linked to both microbial abundance and immune indicators within the correlation network (Figure 8).

**Figure 8:**
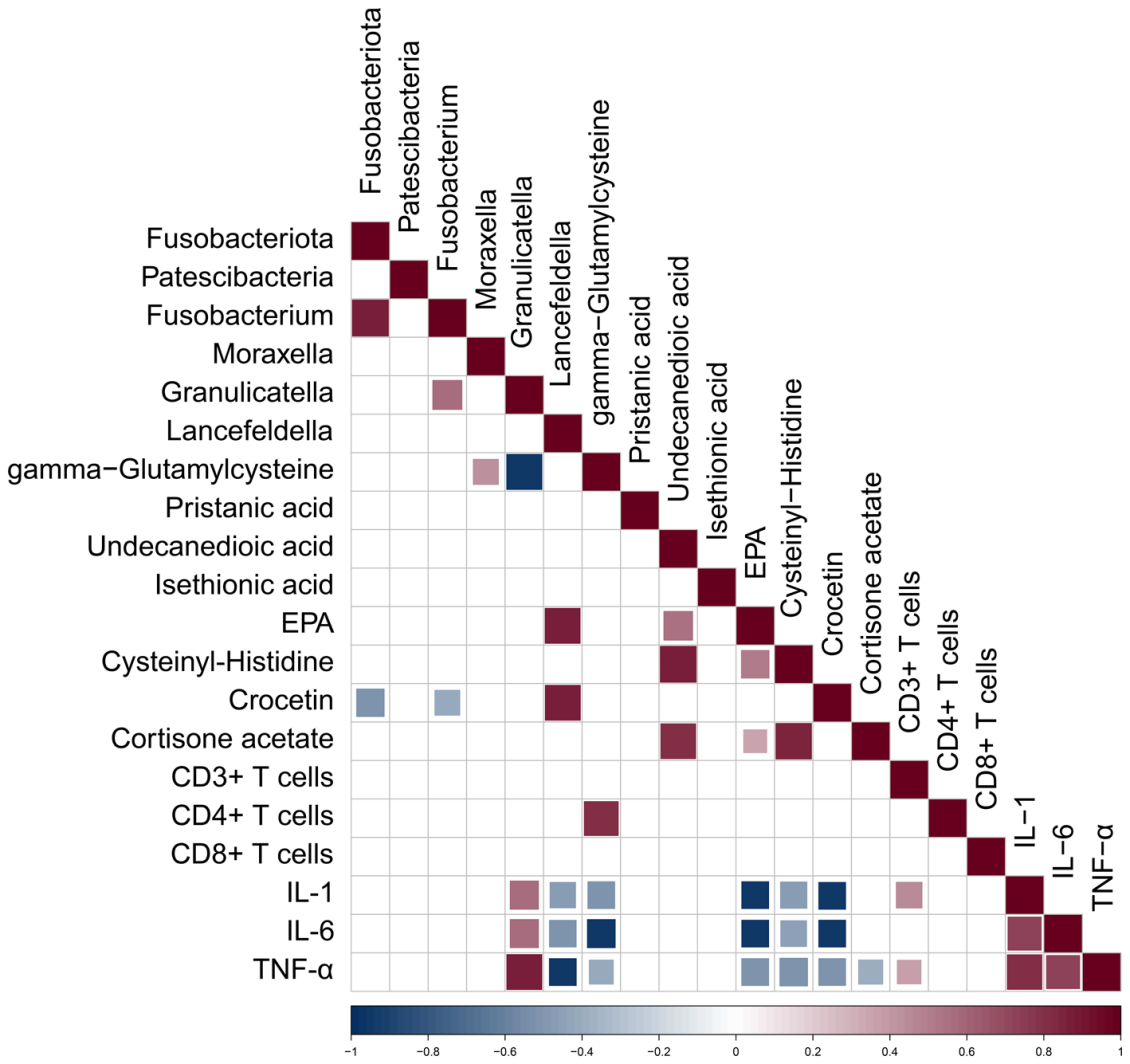
Integrated microbiota-metabolite-immune interaction network in RAU. Correlation networks were constructed based on Spearman correlation analysis between differential microbial taxa, metabolites, and immune-related indicators. Only significant correlations with |*r*| > 0.5 and *p*-value < 0.05 were included in the network. The edge width represents the strength of correlation, and red and blue edges indicate positive and negative correlations, respectively.

## 4 Discussion

Recurrent aphthous ulceration (RAU) is a common chronic inflammatory disorder of the oral mucosa characterized by recurrent ulcer formation and persistent mucosal inflammation. Although its etiology remains incompletely understood, increasing evidence suggests that microbial dysbiosis, metabolic disturbance, and immune dysfunction collectively contribute to disease development (de Barros Gallo et al., 2009; Preeti et al., 2011; Slebioda et al., 2014). In the present study, we performed an integrated multi-omics analysis combining oral microbiome profiling, serum metabolomics, and immune characterization, and identified a coordinated microbiota-metabolite-immune interaction network associated with RAU pathogenesis.

The oral cavity harbors a highly diverse microbial ecosystem that plays a critical role in maintaining mucosal homeostasis (Dewhirst et al., 2010; Kilian et al., 2016). In this study, RAU patients exhibited altered microbial diversity and distinct community structures compared with healthy controls. Although alpha diversity indices showed only a decreasing trend without statistical significance, beta diversity analyses demonstrated a clear separation between groups, indicating that microbial composition was substantially reshaped under disease conditions. These findings are consistent with previous studies reporting that RAU is associated more with compositional dysbiosis than with simple loss of microbial richness (Bankvall et al., 2014; Hijazi et al., 2015). At the taxonomic level, suggestive compositional shifts of specific microbial taxa were observed in RAU patients. Notably, *Fusobacterium*, *Moraxella*, and *Granulicatella* showed trends toward enrichment in the RAU group, whereas *Treponema* and *Lancefieldella* showed trends toward reduction. Fusobacterium has been widely associated with inflammatory oral diseases and epithelial barrier disruption (Brennan and Garrett, 2019; Han, 2015), while *Granulicatella* has been linked to inflammatory biofilm formation and opportunistic pathogenic behavior (Rudney et al., 2001). The observed higher abundance of these genera suggests the potential establishment of a pro-inflammatory microbial microenvironment in RAU. In contrast, the apparent depletion of *Lancefieldella* and *Treponema* may reflect the loss of potentially beneficial commensal bacteria involved in maintaining oral ecological balance. Simper analysis further demonstrated that *Neisseria*, *Prevotella*, and *Haemophilus* were major contributors to inter-group microbial dissimilarity, highlighting that shifts in dominant oral genera may collectively drive disease-associated microbial restructuring (Segata et al., 2012). Importantly, PICRUSt2 functional prediction suggested that the impact of microbial dysbiosis extended beyond taxonomic alterations to functional reprogramming of the microbial community (Douglas et al., 2020). Differential enrichment of pathways related to amino acid metabolism, carbohydrate metabolism, and inflammatory signaling indicated that RAU-associated microbiota may actively participate in host metabolic and inflammatory processes. The increased number of unique predicted functions observed in the RAU group further supports the presence of enhanced functional heterogeneity under inflammatory conditions.

Metabolomic analysis revealed extensive systemic metabolic remodeling in RAU patients. In both negative and positive ion modes, PCA analyses demonstrated clear metabolic differentiation between RAU and healthy controls, indicating that RAU is associated with widespread metabolic perturbation. Differential metabolites were primarily enriched in pathways associated with oxidative stress regulation, lipid metabolism, amino acid metabolism, nucleotide metabolism, and inflammatory responses (Estornut et al., 2024). Among these metabolites, several findings are particularly noteworthy. In the negative ion mode, gamma-glutamylcysteine, pristanic acid, undecanedioic acid, and isethionic acid were significantly altered. Gamma-Glutamylcysteine is a critical precursor of glutathione synthesis and plays an essential role in maintaining intracellular redox balance (Forman et al., 2009; Lu, 2013). Its alteration strongly suggests disruption of glutathione-dependent antioxidant defense systems in RAU. In parallel, undecanedioic acid and pristanic acid are closely related to lipid oxidation and fatty acid metabolism, indicating enhanced oxidative lipid damage and metabolic stress. Moreover, isethionic acid, which is associated with microbial sulfur metabolism, may provide an additional mechanistic link between oral microbial dysbiosis and host metabolic disturbance. In the positive ion mode, metabolites including eicosapentaenoic acid (EPA), crocetin, cortisone acetate, and cysteinyl-histidine dipeptides were significantly altered. EPA is a key ω-3 polyunsaturated fatty acid involved in the biosynthesis of specialized pro-resolving mediators that terminate inflammation and promote tissue repair (Calder, 2010; Serhan, 2014). Reduced EPA-associated signaling may therefore contribute to persistent inflammation and delayed mucosal healing in RAU. Crocetin and cysteinyl-containing dipeptides are closely associated with antioxidant capacity and ROS scavenging (Shah et al., 2021), further supporting the presence of oxidative stress imbalance in RAU patients. Collectively, these metabolic alterations indicate that RAU is characterized not only by inflammatory activation but also by impaired inflammation resolution and defective antioxidant defense. KEGG pathway enrichment analysis further reinforced these observations. In the negative ion mode, pathways associated with cysteine and methionine metabolism, nicotinate and nicotinamide metabolism, and β-alanine metabolism were enriched, highlighting disturbances in redox regulation and amino acid metabolism. In the positive ion mode, pathways related to purine metabolism, taurine and hypotaurine metabolism, and primary bile acid biosynthesis were significantly altered. Purine metabolism is closely linked to inflammatory stress and cellular turnover (Battelli et al., 2018), whereas taurine and hypotaurine metabolism plays an important role in antioxidant and immune regulation (Marcinkiewicz and Kontny, 2014). Network analysis revealed extensive correlations among oxidative stress-related pathways, amino acid metabolism, and inflammatory metabolic pathways, indicating that these metabolic features were closely associated with each other in RAU.

In addition to microbial and metabolic alterations, RAU patients exhibited significant immune dysregulation. Altered proportions of CD4^+^ T lymphocyte subsets indicated imbalance in adaptive immune responses, while elevated IL-1, IL-6, and TNF-α levels reflected a systemic pro-inflammatory state. These findings are consistent with the concept that RAU involves aberrant immune activation and excessive inflammatory responses (Bazrafshani et al., 2002; Lewkowicz et al., 2011; Sun et al., 2006). One of the most important findings of the present study is the integrated correlation analysis linking microbiota, metabolites, and immune parameters. Correlation network analysis demonstrated that inflammatory-associated taxa, particularly *Granulicatella*, were positively associated with pro-inflammatory cytokines, whereas antioxidant-related metabolites such as gamma-glutamylcysteine, EPA, crocetin, and cysteinyl-containing dipeptides were negatively correlated with inflammatory markers. Conversely, *Lancefieldella* showed positive associations with antioxidant metabolites and negative associations with inflammatory cytokines, suggesting a potential protective role. These findings support the existence of a coordinated microbiota-metabolite-immune interaction axis in RAU, consistent with emerging evidence linking oral microbial dysbiosis to host metabolic and immune remodeling (Ning et al., 2023; Zhang et al., 2019). Based on these observations, we propose a conceptual framework in which oral microbial dysbiosis is associated with alterations in inflammatory activation, oxidative stress, glutathione-associated antioxidant pathways, and inflammatory lipid metabolism. These features were also associated with T-cell imbalance, cytokine production, and epithelial injury, suggesting an interconnected relationship among microbial, metabolic, immune, and epithelial alterations in RAU. Further experimental studies are required to determine the causal relationships underlying these associations. In this context, metabolic alterations were associated with both oral microbial dysbiosis and host immune dysfunction. Although oral microbiota and serum metabolites were obtained from different biological compartments, their integrated analysis revealed associations among oral microbial composition, systemic metabolic profiles, and immune responses. However, these findings do not establish causal or directional relationships among these factors, and the potential biological connections between oral microorganisms and circulating metabolites require further investigation.

Several limitations of this study should be acknowledged, including the relatively small sample size and the cross-sectional design, which preclude causal inference and broad generalizability. Importantly, we did not systematically collect information on key lifestyle and clinical confounders, such as detailed dietary habits (including caffeine intake), smoking status, oral hygiene, menstrual cycle phase, and clinical severity parameters (e.g., ulcer number, size, and disease scores). The absence of these data limits our ability to adjust for potential confounding effects on microbiota, metabolite, and immune profiles, and prevents evaluation of associations with disease activity. Although standardized sample collection was performed, residual confounding cannot be excluded, and our metabolomic findings, particularly those related to diet-influenced metabolites, should be interpreted with caution. Future prospective studies integrating comprehensive covariate collection, standardized severity assessments, and longitudinal mechanistic designs are necessary to validate and extend our findings.

## 5 Conclusion

Our findings support an integrated association model in which oral microbial dysbiosis, metabolic alterations, and immune responses are interconnected in patients with recurrent oral ulceration (Figure 9). These coordinated changes were associated with oxidative stress-related signatures, altered lipid metabolism, and immune activation, which may reflect the biological processes involved in mucosal dysfunction. This integrative framework provides new insights into the molecular features of recurrent oral ulceration and highlights potential therapeutic strategies, including modulation of oral microbiota and regulation of metabolic and immune homeostasis, which require further validation in future mechanistic studies.

**Figure 9:**
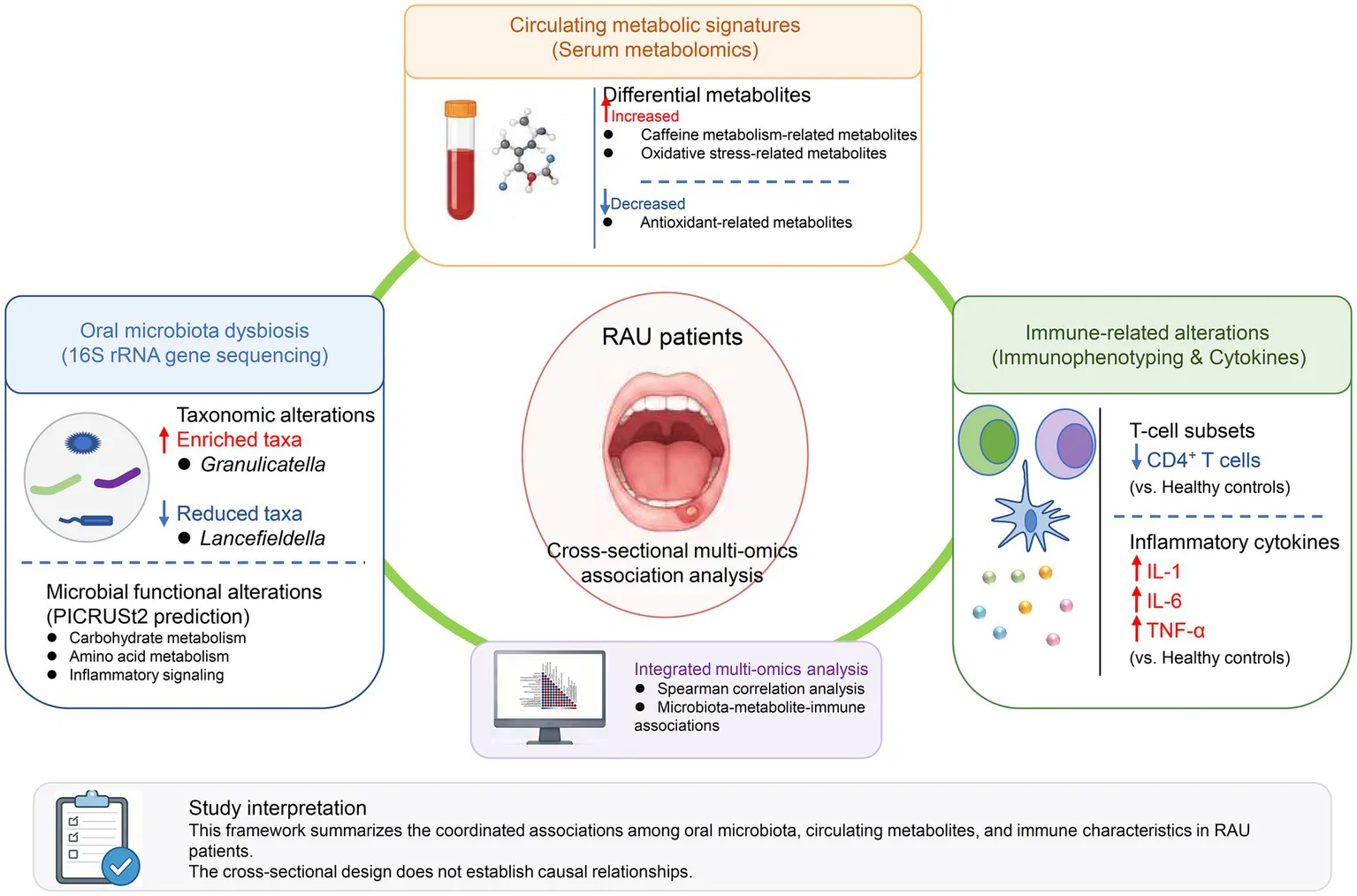
Integrated microbiota-metabolite-immune association profile in RAU. Oral microbial dysbiosis in RAU patients was associated with altered microbial functional profiles and circulating metabolic signatures. These microbial and metabolic alterations were accompanied by changes in immune-related parameters, including T-cell subsets and inflammatory cytokine profiles. The integrated multi-omics analysis revealed coordinated associations among oral microbiota, serum metabolites, and immune characteristics, providing a comprehensive overview of molecular alterations associated with RAU.

## Data Availability

The datasets presented in this study can be found in online repositories. The names of the repository/repositories and accession number(s) can be found below: the raw 16S rRNA gene sequencing data generated in this study have been deposited in the NCBI Sequence Read Archive (SRA) under accession number PRJNA1512584. The processed metabolomics feature matrix, metabolite annotations, and associated metadata are provided in Supplementary Table S3. Raw metabolomics mass spectrometry files are available from the corresponding author upon reasonable request.
